# How perceived social support influences Chinese students’ intention to change majors: a chain mediation model with moderation

**DOI:** 10.3389/fpsyg.2025.1502128

**Published:** 2025-03-26

**Authors:** Zhanning Wang

**Affiliations:** School of Public Administration, Shandong Normal University, Jinan, China

**Keywords:** perceived social support, intention to change majors, SCCT, chain mediation effect, moderation effect

## Abstract

**Introduction:**

Reducing students’ non-interest-oriented and passive intention to change majors is crucial to reducing the risk of changing majors and improving the quality of talent training. Existing research often lacks a coherent framework, which makes the mechanisms by which students’ perceived external environmental factors influence major mobility behaviours unclear. Therefore, this study used Social Cognitive Career Theory (SCCT) to explore the complex relationship between students’ social support and their intention to change their major cognition.

**Methods:**

The paper surveyed students at four universities in Shandong Province, China, in 2024 regarding perceived social support, intention to change majors, mental flow experience, self-efficacy, and major cognition.

**Results:**

(1) In terms of the total effect, students’ perceived social support was significantly negatively correlated with their intention to change majors. (2) In the mediation effect analysis, students’ experience of mental flow in major learning and self-efficacy played an important chain mediation role between perceived social support and the intention to change majors. This explains the internal influence mechanism of the total effect. (3) In the analysis of direct effects, students’ perceived social support was significantly and positively correlated with their intention to change majors. This is contrary to the relatively high proportion of the chain mediation effect, but reflects the current situation of some students’ choice of majors. (4) In the analysis of moderation effects, the relationship between students’ perceived social support and their intention to change majors was positively moderated by major cognition.

**Discussion:**

Students’ intention to change majors decreases when they translate perceived social support into a mental flow experience of major learning and gain self-efficacy for major learning. When the two directly interact, a high level of perceived social support enhances students’ intention to change majors. Students’ major cognition moderates the relationship between perceived social support and mental flow. The main social support providers for students (government, school, family members) should consider focusing on whether support behaviours are translated into internal motivation for students’ major learning, so as to ensure that students make rational major changes.

## 1 Introduction

Choosing a major is a crucial prerequisite for major training, and making a reasonable choice is essential for cultivating high-quality majors (Zhang and Wang, 2014). Because the first choice of major after the college entrance examination is restricted by objective factors such as admission scores and major cognition, it has led to a “mismatch” between students’ learning interests and major training goals. Therefore, many countries and regions have formulated a policy of major mobility, allowing students to “choose a major for a second time.” In recent years, some top universities in People’s Republic of China, such as Shanghai Jiao Tong University (SJTU), Tongji University (TJU), and Wuhan University (WHU), have broken the traditional “high score priority” policy, gradually loosening restrictions on major changes and achieving “zero threshold” major changes, thereby helping students to make up for the regret of their initial major choice and better achieve interest-oriented major talent training (Lin et al., 2011). However, a relaxed policy on major changes will inevitably lead to a “siphon effect” in popular majors, resulting in confusion in major management and an imbalance in talent training across disciplines (Bean, 1988). Current research on student major changes lacks dialectical thinking. A large number of studies suggest that universities continue to lower the threshold for major changes based on students’ learning interests, but ignore the utilitarian choices of students’ major changes.

Although satisfying students’ major learning interests is the main purpose of universities’ major mobility policies, other than self-determined individual closed factors such as students’ major interests and major identity (Scheunemann et al., 2022), family support, peer support, teacher support, and other environmental open factors also have an impact on student’s intention to change majors (Hill and Torres, 2010; Rayle and Chung, 2007). Part of the research suggests that a high level of perceived social support reduces the intention to change majors (Wang and Eccles, 2013). While others suggest that the higher the perceived social support, the higher the intention to change majors (Mishra, 2020). Compared to students’ self-determination, social factors have a more complex impact on their intention to change majors and need to be analysed in greater depth.

Given the complexity and unpredictability of perceived social support, Social Cognitive Career Theory (SCCT) is well-suited for studying students’ intention to change majors. It links individual psychological factors with external environmental factors (Liu et al., 2020), offering a comprehensive framework for predicting this intention. Therefore, based on SCCT, this research focuses on two questions: (1) Does perceived social support affect students’ intention to change majors? (2) How does perceived social support influence students’ intention to change majors?

Students’ major cognition is a unique feature of “major change,” which generally takes place at the end of the first year of study (Yu and Zhang, 2021). At this time, major cognition provides students with a framework for understanding and evaluating the risks and opportunities of major change, which is also an important basis for the formulation of major change policies (Abercrombie et al., 2022). Different students have different major cognitions, and the role of social support in students’ major change behaviour also changes (Forsblom et al., 2022; Jia and Cheng, 2024). When students are in a state of high major cognition, perceived social support, whether or not it is converted into internal motivation for students’ major learning, will lead to an increase in students’ intention to change majors. However, when students are in a state of low major cognition, perceived social support converted into internal motivation will help students reduce their intention to change majors. This will provide targeted suggestions for the improvement of future major change policies.

The potential marginal contribution of this paper lies in the application of the SCCT to students’ psychological flow and self-efficacy in major studies as mediating variables. This approach links students’ perceived social support in an open environment with a closed environment, which fills the gap in current research on the mechanism of the impact of students’ perceived social support on their intention to change majors. It also addresses the lack of practical significance in current research by studying from the initial goal of major change policies-maximising the benefits of major changes by studying in a way that is guided by students’ interests (State Council of the People's Republic of China, 2017). Building on the mediating effect model in this study, major cognition is introduced as a moderating variable, considering the characteristics of second major selection, to more comprehensively explain the impact of students’ perceived social support on their intention to change majors.

## 2 Literature review

There are two factors that affect college students’ intention to change majors: one is based on their subjective feelings in a closed situation, and the other is based on their perceived social support in an open situation. In a closed situation, there is a significant correlation between students’ intention to change majors and their subjective feelings about the major, which has been widely studied and confirmed (Respondek et al., 2017; Tomlinson and Jackson, 2021). Students are less likely to change their major when they have a higher interest in the major (Scheunemann et al., 2022), clearer goals (Permatasari et al., 2021), more confidence in employment prospects, and are more satisfied with the major (Allen and Zhang, 2015).

Compared with studies on students’ subjective feelings, the mechanisms that affect students’ intention to change majors in an open situation are more complex and have attracted widespread attention from scholars in recent years. The open situation focuses on students’ external social support. Students’ social circles are relatively single, and in recent years have mainly included three dimensions: family support, school support, and peer support (Li et al., 2018). The impact of these three dimensions on students’ intention to change majors is controversial. Family and teachers provide students with emotional support and academic guidance, which increases students’ sense of belonging to their majors and thus reduces their intention to change majors (Tinto, 2017; Wang and Eccles, 2013). Peer support can help students improve their psychological resilience when facing pressure, thereby reducing their intention to change majors (Tinto, 2017). However, social support does not always have a positive impact. Family pressure support and teacher rejection support can increase students’ intention to change majors (Roksa and Kinsley, 2019; Rayle and Chung, 2007), while students’ sharing negative emotions about their majors with peers can increase their intention to change majors (Hill and Torres, 2010). There is also debate about combining students’ family support, teacher support and peer support into an overall variable of perceived social support (Chen et al., 2023; Mtshweni, 2024).

A single study of open and closed situations lacks practical significance. The SCCT states that people are the product of dynamic interactions between external environmental factors and internal subjective factors, so social support in open situations and students’ motivation to study professionally in closed situations should be systematically analysed (Nguyen et al., 2025). Previous studies have used students’ psychological capital (Hu, 2021; Luthans et al., 2007), employment confidence (Wang, 2024; Betz et al., 1996) and emotional regulation ability (Gross, 2002) as mediating variables of perceived social support and intention to change majors, but few studies have focused on the mediating role of students’ mental flow experience and self-efficacy in major learning. A mental flow is an experience of complete focus on a task, the highly concentrated attentional experiences possessed by an individual (Csíkszentmihályi, 1975). Rumann and Hamrick (2010) showed in their study that social support can redefine one’s sense of self-learning and mental flow experience. When students are immersed in a long-lasting mental flow experience during their major studies, their major cognition will change, which in turn affects their intention to change majors. Self-efficacy in major learning is the result of acceptance of learning in flow, and both represent the main internal motivation of students in major learning behaviour. Therefore, as a representative factor of the closed environment, it has a strong persuasive power as an intervening variable (Guyotte et al., 2019; Williams and Roberts, 2023).

In studies on the mechanisms by which students perceive social support and their intention to change majors, several studies have focused on the moderating effect. Findings from research have shown that students’ psychological resilience (Connor and Davidson, 2003) and the inherent characteristics of the campus environment (Tinto, 1987) moderate the correlation between students’ perceived social support and their intention to change majors. It is not difficult to find that there are currently few studies focusing on the particularity of students’ second major change. The second major change is a major choice based on a certain major learning foundation, which is one of the important differences from the first major choice.

In summary, most of the current research has reached a consensus on the influence of students’ subjective factors on their intention to change majors and partially explored the pathways between external perceived social support and their intention to change majors. The results of the findings confirm that students’ positive major effect significantly reduces their intention to change majors while revealing the complex mechanism of the role of external perceived social support on the intention to change majors. This provides a better foundation for this study and has a high reference value. However, the existing research has the following shortcomings: (1) the mechanism of the impact of perceived social support on students’ intention to change majors is controversial. (2) The pathways of students’ perceived social support and intention to change majors have not been fully investigated. Current research methods mostly use Regression toward the mean, simple mediating effect analysis, and simple moderating effect analysis, and do not pay attention to the specificity of major mobility, which has limitations to the in-depth exploration of the path. (3) Few studies have explored the mutual transformation between students’ closed and open environments. Although there is currently a theory that combines the two for research, it is less commonly used in studies of students’ major changes, which is unrealistic in the Chinese context.

## 3 Theoretical framework

**Figure 1:**
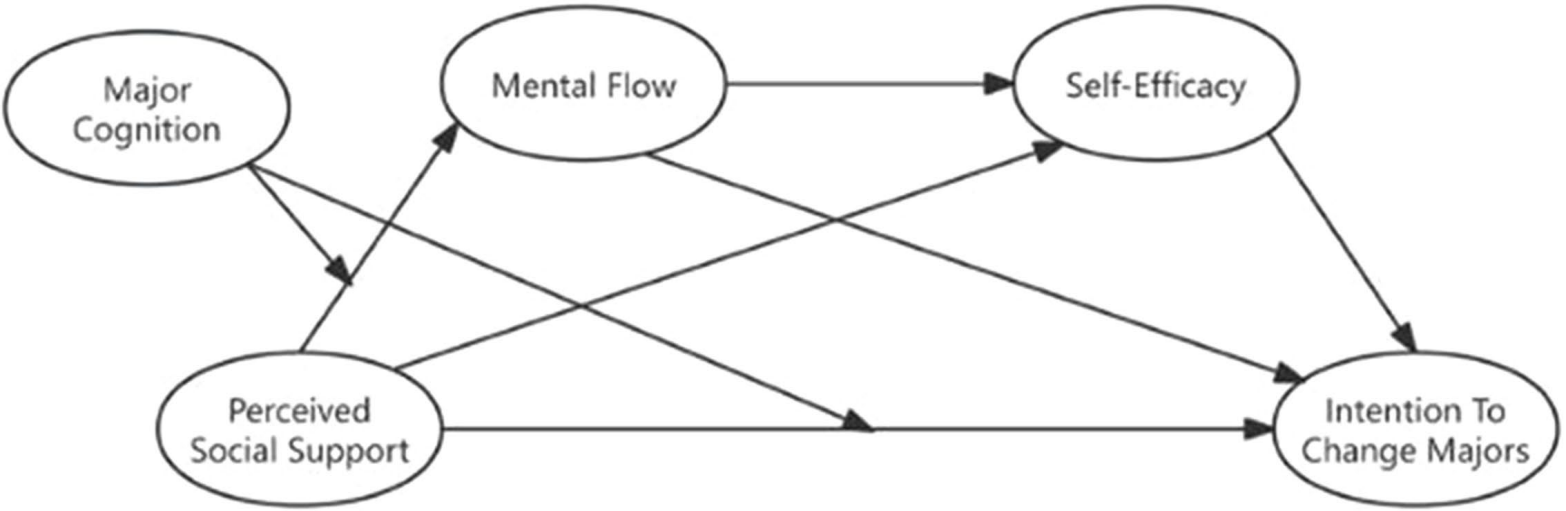
Research model for the social support, flow state, self-efficacy, major cognition, and intention to change majors.

SCCT states (Lent et al., 1994) that interests and hobbies will have the greatest impact on major choices under favorable social and environmental conditions, enabling people to pursue their interests. But many people are prevented from pursuing their interests unimpeded and with the full support of others. The choices of these people are constrained by experiences such as economic needs, family pressures, or educational restrictions, as well as being more in alignment with the actual needs faced by today’s diverse society. Accordingly, SCCT links a person’s social support and positive experiences gained from engaging in a certain behaviour to self-recognition. Specifically, when individuals feel positive social support, they will have a higher degree of behavioural experience or self-recognition, thereby increasing the likelihood that the individual will choose or persist in this thing or behaviour. Therefore, as shown in Figure 1, this study introduces the SCCT perspective to examine the relationship between students’ perceived social support and their intention to change majors.

The social support that people receive as socialized groups strongly governs behavioural intentions (Kingsford-Smith et al., 2024). The constraining force of the environment is coercive and deterministic, but a direct intervention that fails to persuade the individual’s intention will increase the individual’s rebelliousness and produce counterproductive effects (Marquardt et al., 2022). Relevant studies have pointed out that leaders who rely solely on rules and terms that have nothing to do with employees’ interests to supervise their employees’ productivity, employees’ work commitment and motivation will be significantly reduced, and they may even develop a tendency to leave their job (Endalcachew et al., 2023). Where students’ perceived social support fails to translate into an individual’s mental flow in their major learning, and instead acts directly on the intention to change majors, this can increase rebelliousness and self-reflection, and their intention to change majors can increase. Therefore, the following hypotheses are proposed in this study:

H1: There is a positive correlation between students’ perceived social support and the direct effect of their intention to change majors.

Self-efficacy is people’s subjective judgment of their ability to engage in a behaviour successfully (Bandura, 1971). It is a subjective assessment of an individual’s ability and internal motivation that drives people to perform a variety of activities. A mental flow is often associated with self-efficacy (Ye, 2024). Bandura (1986) argues that factors such as an individual’s experience of success and failure, alternative experiences, emotional and physiological states, and verbal persuasion have a significant impact on self-efficacy. Students’ perceived social support, on the other hand, refers to the recognition and motivation that students receive from their families, teachers, and peers (Wang et al., 2014).

SCCT suggests that a person’s perceived social support alters the person’s psychological state, which in turn affects the personal sense of self-efficacy, thereby altering career goals (Buthelezi et al., 2009). It has also been proven that when individuals have a high level of social support capital, external disruptive factors are reduced, making it easier for individuals to enter the mental flow (Huang and Zeng, 2010). Accordingly, the phenomenon that students who have a higher mental flow receive positive emotional feedback, leading to an increase in personal self-efficacy, cannot be demonstrated during the initial major decision-making process. The phenomenon may need to be clarified after a certain period of major study and major cognition (McLean, 2016). When students receive more encouragement and cues for their majors from their families, peers, and faculty, students’ resistance to their majors diminishes and their mental flow during their majors increases (Demir and Leyendecker, 2018). The mental flow has been shown to increase an individual’s efficiency and motivation in completing tasks (Ye, 2024). So with effective major learning, students will increase their confidence in their major learning, thereby increasing their self-efficacy, and their intention to change majors will decrease when they have high levels of self-efficacy. Lin (2023) is research proves this. However, when students cannot obtain sufficient support from the social environment or even are ostracised, they will lose their motivation to learn in their major, which will reduce the mental flow of students’ major learning, and then decrease students’ self-efficacy (Razali et al., 2022), which will make students reduce their commitment to their major and create their intention to change majors. Therefore, the following hypothesis is proposed in this study:

H2: Students' mental flow of major learning and self-efficacy play a chain mediating role in students' perceived social support and intention to change majors.

The students’ second choice of major is usually made after the completion of the first year of university courses, and unlike the first choice of major, the second choice of major has a certain amount of major learning experience (Silver, 2023), so this differs from the level of major cognition of the first choice of major. SCCT also points to the importance of an individual’s subjective perception of things, as a factor independent of perceived social support (Lent et al., 1994). Some studies have shown that individuals’ perceptions of things are shaped by different channels of information, and when people have not been exposed to and experienced something, they mainly rely on social experience and the opinions of others to make perceptions, which usually results in cognitive bias (Mugg and Khalidi, 2021). Individuals who have been exposed to things personally will have individual subjective perceptions that exist independently of social environmental biases, at this time social environments will have a significantly lower impact on individuals (MacLeod and Mathews, 2012). Therefore, it can be speculated that in the direct effect, when students have positive major cognition, the effect of students’ intention to change majors and perceived social support may be enhanced. Conversely, when students have negative major cognition, the effect of perceived social support and intention to change majors will be weakened.

Self-Determination Theory (SDT) emphasizes that individuals’ high awareness of their behaviour affects their intrinsic motivation, which in turn motivates individuals to achieve a state of flow during behavioural activities and complete tasks better (Zhou et al., 2022). Research has also shown that individuals with a high level of awareness and subjective experience often use self-awareness as the main reference factor for making choices (Dong et al., 2021). In addition, students’ major cognition can affect their perception of social support (Abercrombie et al., 2022). If students are clear about their learning needs and have a good understanding of their major, they are more able to effectively obtain support from others and turn this support into motivation for learning, enhancing their mental flow experience. For example, students may ask their tutors or peers questions more purposefully, thereby obtaining more targeted help (Yinghua and Tingting, 2021). Therefore, although external support is powerful, an individual’s experience of mental flow is also influenced by their personal perceptions and feelings. At this point, the perceived social support can only be achieved through coercion and external control, but the impact on an individual’s mental flow experience is weakened or even negative. When students change their major as their second choice, they usually have a certain degree of major cognition and learning experience (Yu and Zhang, 2021). When students have a high degree of major cognition, their major learning mental flow experience is mainly influenced by their personal perception of the major. However, when students have a low level of major cognition, their major learning experiences are similar to the role of the external environment when they first chose their major, and are more influenced by family, peers and teachers. Therefore, this study proposes the following hypothesis:

H3: The degree of students’ major cognition plays a significant moderating role in the chained mediating path and the direct effect path.

## 4 Materials and methods

### 4.1 Research procedure and participants

In order to reflect the geographical differences in higher education in China, the study selected four public comprehensive universities in Shandong Province. All four universities recruit students from all over the country to avoid selection bias in the admissions process. First, five major types of majors were selected based on the nature of the major: liberal arts, science, and art. The willingness of students majoring in arts, science and art to change majors will differ (Yu, 2015), so we will proportionally select two arts majors, two science majors and one art major from the three main majors. These were biological science majors, psychology majors, labour and social security majors, public administration majors, and art management majors. Then, a random sample was taken based on the proportion of students enrolled in comprehensive evaluation, students enrolled in specialised streams, and students enrolled in general programmes in the country over the years. The selection of survey respondents was based on specific inclusion and exclusion criteria: (1) Only students who were enrolled on the day of the survey were included, and those who had applied for leave of absence or suspended their studies were excluded; (2) Only undergraduate students were considered, and those who had made a second major change and those who had graduated were excluded, because the policy on major changes in most Chinese universities stipulates that the second choice of major is made while the undergraduate student is still enrolled.

The questionnaire was administered at the beginning of the second semester of the academic year, taking into account the fact that major changes at the university usually take place at the beginning of the semester and students’ major cognition, in April 2024. Data was collected using a self-administered questionnaire. I issued thank-you letters to the students who participated in the survey and paid them accordingly based on the quality of the questionnaire. After trained investigators provided guidance on completing the questionnaire, they reviewed and collected the questionnaires after ensuring that they were correct. A total of 451 questionnaires were distributed for the study, and questionnaires with incomplete answers or logical errors were considered invalid. After excluding the invalid questionnaires, a total of 392 valid responses were collected, for an effective rate of 87%. According to Hair et al. (2018), the optimal ratio of sample variables in a questionnaire survey should exceed 20:1. Therefore, the sample in this study exceeds the required minimum level (392:5).

### 4.2 Measures

A five-level Likert scale was used in all of this study, with standardized procedural scales for perceived social support, major cognition, mental flow, self-efficacy, and the student’s intention to change majors selected and adapted, respectively.

#### 4.2.1 Perceived social support

The Perceived Social Support Scale (PSSS) developed by Zimet et al. (1987), was used and the scale has demonstrated reliability and validity in the Chinese context. The scale consists of 6 question items, and the higher the mean of the total score, the stronger the students’ perceived social support. The total internal consistency coefficient of the scale in this study was 0.890. The average variance extracted (AVE) is 0.615.

#### 4.2.2 Major cognition

There are fewer current quantitative studies analysing the degree of major cognition of students in higher education, and Yanqi Wang’s Major Cognition Scale (Yanqi, 2022) for Students Majoring in Chinese Language was selected and adapted with relevant questions in accordance with the theme of this study. Since the original scale involved questions related to the Chinese language, which is inconsistent with this study, the related questions were deleted. The adapted scale consisted of three items, which were measured in three aspects: major cognition of major training mode, knowledge of major knowledge and theories, and cognition of major competence and literacy. The higher the overall mean, the stronger the students’ major cognition. The overall internal consistency coefficient of the scale in this study was 0.901. The AVE is 0.710.

#### 4.2.3 Mental flow

We chose the Heart Flow State Scale (FSS) developed by Jackson and Marsh (1996). In this study, I followed a strict standard scale translation process, including initial translation by bilingual experts, back-translation by independent translators, and expert review by a panel of language experts, and adapted the scale according to the students’ major studies, and a total of four question items, with the higher the overall mean value, the stronger the students’ mental flow during their major studies. The overall internal consistency coefficient of the scale in this study was 0.916. The AVE is 0.540.

#### 4.2.4 Self-efficacy

For the measurement of self-efficacy, I chose the Self-Efficacy Scale compiled by Henson (Henson, 2001), and formed the Chinese version of the major Self-Efficacy Scale for College Students after following a rigorous standard scale translation process and initial translation by a bilingual expert, back-translation by an independent translator, and review by a language expert. After eliminating items that might have been cross-cutting with concepts of students’ mental flow of their major learning, I kept a total of Seven items that were retained after eliminating the items that might intersect with the concept of students’ experience of major learning mindstream. The questionnaire was scored on a 5-point Likert scale. The higher the mean total score, the stronger the student’s identification with the major. The total internal consistency coefficient for the scale in this study was 0.960. The AVE is 0.580.

#### 4.2.5 Intention to change majors

Compared with the previous three, the variable of intention to change majors has a strong degree of operationalization, and the major intention to change majors in this study mainly refers to the change of majors during undergraduate study. To ensure the rigor of sample sampling, three representative questions were selected, which were ‘I hope to change my major’, ‘I hope to change my major so that I can engage in a job unrelated to my original major in the future’, and ‘I would like to change my major so that I can enroll in a graduate school in another major in the future’. The higher the mean of the total score, the stronger the intention to change majors. The internal consistency coefficient of the scale in this study was 0.851. The AVE is 0.633.

### 4.3 Descriptive statistics of the scale factor

Table 1 shows the mean, standard deviation, skewness and kurtosis of the factors. Skewness describes the symmetry of the data distribution. For a normal distribution, the skewness value should be close to 0. Kurtosis describes the steepness of the data distribution and the degree of concentration compared to a normal distribution. The kurtosis value of a normal distribution is 3. A kurtosis value above this indicates a heavier or lighter tail of the data distribution. According to the calculation, the perceived social support has a kurtosis of 0.468 and a skewness of −0.706; major cognition has a kurtosis of −0.153 and a skewness of −0.325; mental flow has a kurtosis of 0.535 and a skewness of −0.696; self-efficacy has a kurtosis of 0.001 and a skewness of −0.561; intention to change majors has a kurtosis of −0 0.609, and the skewness is 0.491. The skewness and kurtosis of each scale meet the criteria for normal distribution, indicating that the distribution of the data in this scale is approximately normal. It can be considered that univariate normality is met and suitable for normal testing or statistical analysis methods based on normal distribution.

**Table 1:** Descriptive statistics of the scales.

	Mean	SD	Skewness	Kurtosis
1. Perceived Social Support	3.99	0.75	−0.71	0.47
A1. Share joys and sorrows in major learning with teachers.	3.95	0.95	−0.85	0.33
A2. Major help can be obtained from peers	3.96	0.95	−1.00	0.95
A3. Can talk to family about major learning challenges	3.94	1.06	−0.86	0.01
A4. Share joys and sorrows in major learning with friends.	4.15	0.84	−1.01	1.26
A5. Teachers care about major learning	3.92	0.91	−0.75	0.37
A6. Discuss problems with peers	4.03	0.89	−0.98	0.99
2. Major recognition	3.49	0.95	−0.33	−0.15
B1. Familiar with the model of my major training	3.46	1.05	−0.34	−0.49
B2. Familiar with major knowledge and theoretical knowledge	3.48	1.00	−0.32	−0.31
B3. Major competence and core literacy in the field of specialization	3.54	1.03	−0.42	−0.29
3. Mental Flow	3.79	0.87	−0.70	0.54
C1. Enjoy learning about the major	3.85	0.96	−0.81	0.39
C2. In the process of setting up the learning objectives for major knowledge, there is no distraction.	3.81	0.97	−0.73	0.18
C3. Willing to do their best to learn their major well	3.99	0.95	−0.85	0.42
C4. Staying in an optimal state of arousal during major classes	3.50	1.01	−0.28	−0.49
4. Self-Efficacy	3.57	0.95	−0.56	0.00
D1. Happy to work in a major related to their studies	3.73	1.11	−0.71	−0.13
D2. Have accepted this major in their hearts	3.72	1.08	−0.64	−0.22
D3. Generally enjoy the major	3.65	1.08	−0.54	−0.37
D4. Read books related to the major frequently	3.01	1.15	0.13	−0.83
D5. Think that personality matches the major	3.52	1.12	−0.42	−0.60
D6. Think that this major can reflect your strengths	3.72	1.04	−0.76	0.22
D7. Very confident about the future of the major	3.62	1.18	−0.54	−0.56
5. Intention To Change Majors	2.69	1.16	0.29	−0.77
E1. Hope to be able to transfer majors	2.41	1.40	0.58	−0.96
E2. When graduating and looking for a job, wanting to choose a job in a different field, choosing a job unrelated to the field of study	2.83	1.28	0.16	−0.96
E3. Postgraduate students will take exams in other majors	2.29	1.29	0.67	−0.71

### 4.4 Scale confirmatory factor analysis and structural validity

Structural equation model fitting was performed on the hypothetical model. The *χ*^2^/df of the intermediary benchmark model was 2.122, RMSEA was 0.054, CFI was 0.966, IFI was 0.967, TLI was 0.961, and SRMR was 0.045, all of which met the standards. After introducing major identity as an adjustment variable, the model indicators (*χ*^2^df = 2.259, RMSEA = 0.057, CFI = 0.967, IFI = 0.967, TLI = 0.962, SRMR = 0.043) also meet the standards, indicating that the research hypothesis model fits the data well. The fit indices of the four-factor model, five-factor model, and other competing models are shown in Table 2. In addition, CFA can be used to assess the model’s degree of fit and indirectly to assess the Sample Size (Maccallum et al., 1996). When performing CFA, Sample Size is important for obtaining stable and reliable results. A confirmatory factor analysis can be used to determine whether the sample size for this study is sufficient. In the Bartlett Test of Sphericity, the significance of the variables is <0.001. Generally speaking, when the result of the Bartlett Test is <0.05, the sphericity hypothesis is rejected, there is a correlation between the original variables, and it is suitable for factor analysis. In the Kaiser-Meyer-Olkin tests, the variable KMO is 0.745. Generally speaking, when the KMO statistic is >0.5, it can be seen that there is not much difference in the degree of correlation between the variables, and the above data indicates that the variables have the same variance.

**Table 2:** Confirmatory factor analysis.

Fitness Model	c2	df	c2/df	RMSEA	CFI	IFI	TLI	SRMR
Benchmark model (four-factor model)	370.519	164	2.259	0.057	0.967	0.967	0.962	0.043
Five factors model	466.901	220	2.122	0.054	0.966	0.967	0.961	0.045
Three-factor model	1821.117	227	8.023	0.134	0.783	0.784	0.758	0.126
Two-factor model	2104.591	229	9.19	0.145	0.745	0.746	0.718	0.134
Single-factor model	2942.043	230	12.791	0.174	0.631	0.632	0.594	0.140

### 4.5 Data analysis

This study used SPSS 26.0 statistical software to conduct descriptive statistics on the sample. Structural Equation Modelling (SEM) software AMOS was used to conduct path analysis of chain mediation and moderating effects to explore the influence mechanism of perceived social support and intention to change majors. The Bootstrap method of bias-corrected percentile was used to test the mediating and moderating effects. The statistical significance of the mediating variables was set at 95% Confidence Intervals (CI). A hypothesis would be supported if 0 is excluded in the CI or the CI for the indirect effects do not straddle 0 (Hayes and Rockwood, 2017).

In addition, we also tested the risk of multiple collinearity between the intention to change majors and perceived social support, mental flow experience, self-efficacy, and major cognition. The results of the collinearity statistics showed that there was no potential for multiple collinearity with a variable inflation factor (VIF) of 1.310 for the perceived social support variable, a VIF of 2.504 for the mental flow experience variable, a VIF of 2.400 for the self-efficacy variable, and a VIF of 1.646 for the major cognition variable (the criterion value for VIF is <5).

## 5 Results

### 5.1 Descriptive statistics

With regard to the gender, household registration, and major categories of the survey respondents, the proportions are consistent with the distribution ratios of the higher education statistics published by the Ministry of Education of the People's Republic of China (2022). When most of the survey respondents’ major choices are made independently, and the default choice is an “interest-based” major choice, rather than a “utilitarian” major choice, it can be seen that the proportion of “interest-based” major choices is still higher than the that of “utilitarian” major choice in the initial major choice of current students. See Table 3 for more details.

**Table 3:** Sample descriptive statistics.

Type	*N*	Percentage (%)	Effective percentage (%)	Cumulative percentage (%)
Gender				
Male	94	23.98	23.98	23.98
Female	298	76.02	76.02	100.00
Household				
Urban	174	44.39	44.39	44.39
Rural	218	55.61	55.61	100.00
Type of major				
Humanities and Social Sciences	180	45.92	45.92	45.92
Science and Engineering	144	36.73	36.73	82.65
Arts and Sports	68	17.35	17.35	100.00
Major Choice				
Individual choice	246	62.76	62.76	62.76
Parent’s wishes	29	7.40	7.40	70.16
Institutional advice	51	13.01	13.01	83.17
Transferring majors	66	16.84	16.84	100.00

### 5.2 Investigating the mediating effect

**Figure 2:**
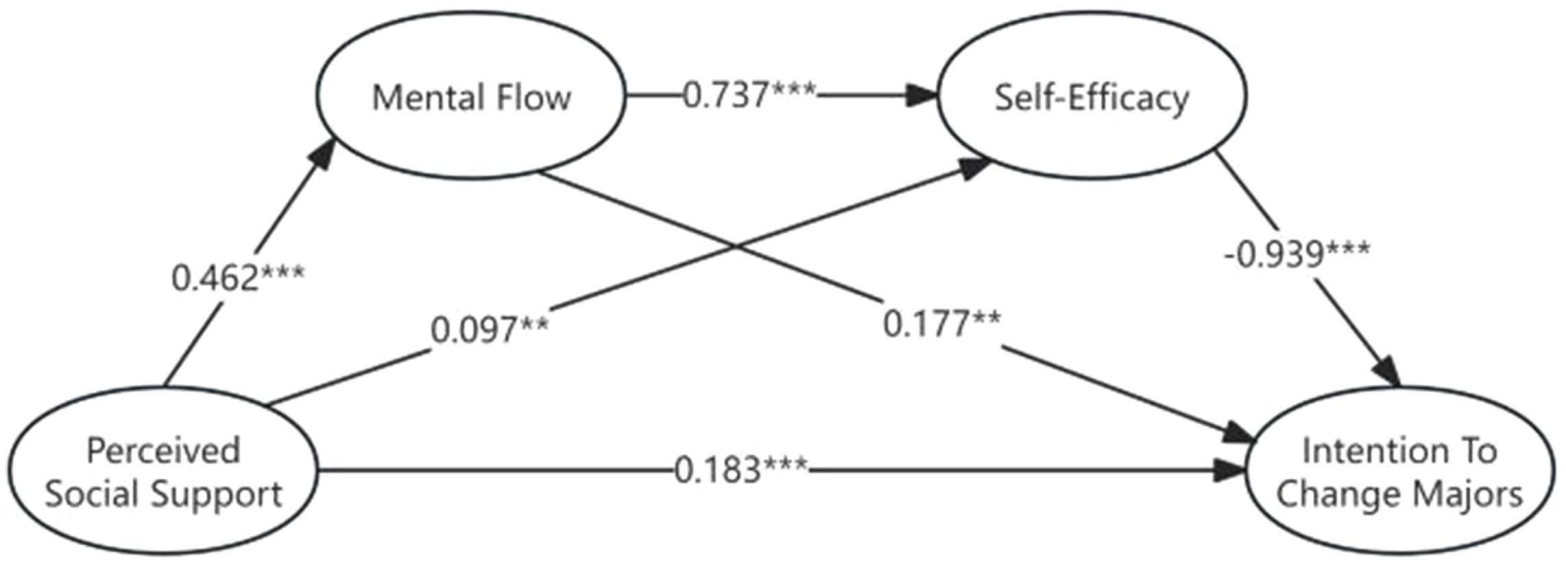
Regression and chain mediation models.

According to the results of the path analysis (Figure 2), firstly, the total effect of students’ perceived social support and their intention to change majors was significant, and the two showed a negative correlation (β = −0.166, *p* = 0.004). In the detailed path analysis, it can be seen that the results of the total effect were mainly provided by the chain mediation effect path. In the chain mediation path, increasing the perceived level of social support can improve students’ experience of mental flow in their major studies (β = 0.462, *p* < 0.001), thereby enhancing their self-efficacy in major studies (β = 0.737, *p* < 0.001), and ultimately having a negative impact on their intention to change majors (β = −0.939, *p* < 0.001). In addition to the chain mediation effect, increasing students’ perceived social support can also directly reduce their intention to change majors by increasing their self-efficacy in major learning (β = 0.097, *p* = 0.021). However, the path coefficient of perceived social support and major self-efficacy is small, and it contributes less to the total effect.

Interestingly, the direct effect of students’ perceived social support on their intention to change majors was the opposite of the total effect. When students’ perceived social support directly affected their intention to change majors, the two showed a significant positive correlation (β = 0.183, *p* < 0.001). In the mediation path with the experience of mental flow as a single mediating variable, perceived social support could produce a significant positive correlation through students’ experience of mental flow (β = 0.177, *p* = 0.011). This shows that perceived social support enhances students’ intention to change majors when students’ perceived social support cannot be translated into self-efficacy for major learning.

To ensure the reliability of the analysis of the mediating effect, Bootstrap was used to test the mediating effect. The Bootstrap test further showed that the mediating effect was generated by three mediating chains. The first chain of mediating effects is that perceived social support (PSS) significantly affects the intention to change majors (CM) through mental flow (MF) and self-efficacy (SE). The confidence intervals of the bias-corrected and percentile tests do not include 0. The second chain of mediating effects is that self-efficacy significantly affects the intention to change majors through perceived social support. The confidence intervals of the bias-corrected and percentile tests do not include 0. The third article is that the mediating effect of the mental flow in perceived social support and intention to change majors is significant, and the confidence intervals of the bias-corrected and percentile test results do not include 0. In the test of the mediating effect ratio, the total effect is offset by the direct effect, because the path direction of the direct effect is inconsistent with that of the indirect effect. Therefore, the total indirect effect accounts for 260.9% of the total effect, the total indirect effect is significant, and the indirect effect actually contributes more influence than the total effect, so H2 is proved.

The direct effect of the model is significant, with a bias-corrected 95% confidence interval of [0.157, 0.386] and a percentile 95% confidence interval of [0.153, 0.381]. Both confidence intervals exclude 0, which means that H1 is established.

### 5.3 Investigating the moderating effect

**Figure 3:**
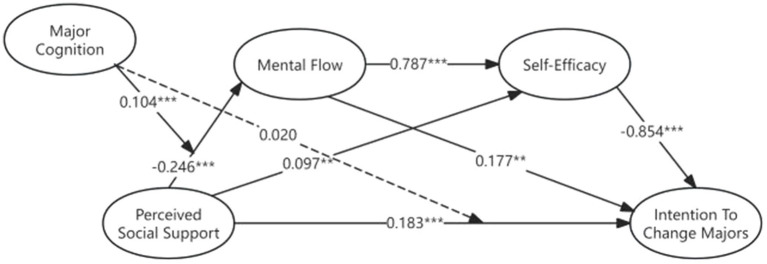
Path diagram of the moderating effect.

In order to test the moderating effect of students’ major cognition (MC) on perceived social support, a new SEM model was established. According to Ping (1995), the path coefficient of the interaction term (perceived social support and major cognition) needs to be calculated based on the effects of each observed variable in the linear model. Then, the interaction term was added to the model, and a direct relationship between the interaction term and the mental flow was set. The analysis results in Figure 3 show that major cognition fits well in the chain-mediated path. The addition of the interaction term resulted in a better fit of the model. At the same time, the interaction term between perceived social support and major cognition was significantly positively correlated with mental flow (β = 0.103, r < 0.001).

In the chain-mediated path of perceived social support and intention to change majors, the 95% BootCI confidence interval for the low level (-1SD) of major cognition is [0.055, 0.536], and the confidence interval does not include 0. The moderating effect of this path is significant, indicating that when major cognition is at a low level, perceived social support has a stronger effect on major mobility through flow experience and self-efficacy. The 95% BootCI confidence interval for the mean level of major cognition is [0.008, 0.426], which does not include 0. The moderating effect of this path is significant, indicating that when students’ major cognition is at a normal level, the relationship between perceived social support and intention to change majors through mental flow and self-efficacy can be observed, and this relationship is universal in the overall sample. The 95% BootCI confidence interval for the high level (+1SD) of this path is [−0.042, 0.320], which includes 0, indicating that the moderating effect of this path is not significant. High levels of students’ major cognition weaken the effect of perceived social support because major cognition provides sufficient “protection” or ‘substitution.’ In summary, the results show that, except for the high level of the chain-mediating effect, which is not significant, the mean value and the low level are both significant at the three different levels, indicating that major cognition has a significant moderating effect in the chain-mediating effect. But in the direct path between perceived social support and intention to change majors, major cognition cannot play a significant moderating role (β = 0.020, r = 0.101). See Table 4 for more details.

**Table 4:** Results of the mediating effect.

Effect types	Point estimate	Product of coefficients		Bootstrapping			
				Bias-corrected 95% CI		Percentile 95% CI	
		SE	Z	Lower	Upper	Lower	Upper
Indirect effect							
SS → FS → SE → CM	−0.425	0.072	−5.903	−0.570	−0.320	−0.552	−0.314
SS → SE → CM	−0.121	0.061	−1.984	−0.220	−0.022	−0.233	−0.031
SS → FS → CM	0.102	0.042	2.429	0.036	0.177	0.034	0.172
TIE	−0.444	0.075	−5.920	−0.570	−0.324	−0.581	−0.332
Direct effect							
DE	0.228	0.057	4.000	0.149	0.341	0.143	0.326
Total effect							
TE	−0.216	0.082	−2.634	−0.344	−0.072	−0.361	−0.087
Percentage							
TIE/TE	2.059	2.328	0.884	1.479	4.912	1.448	4.163

**Figure 4:**
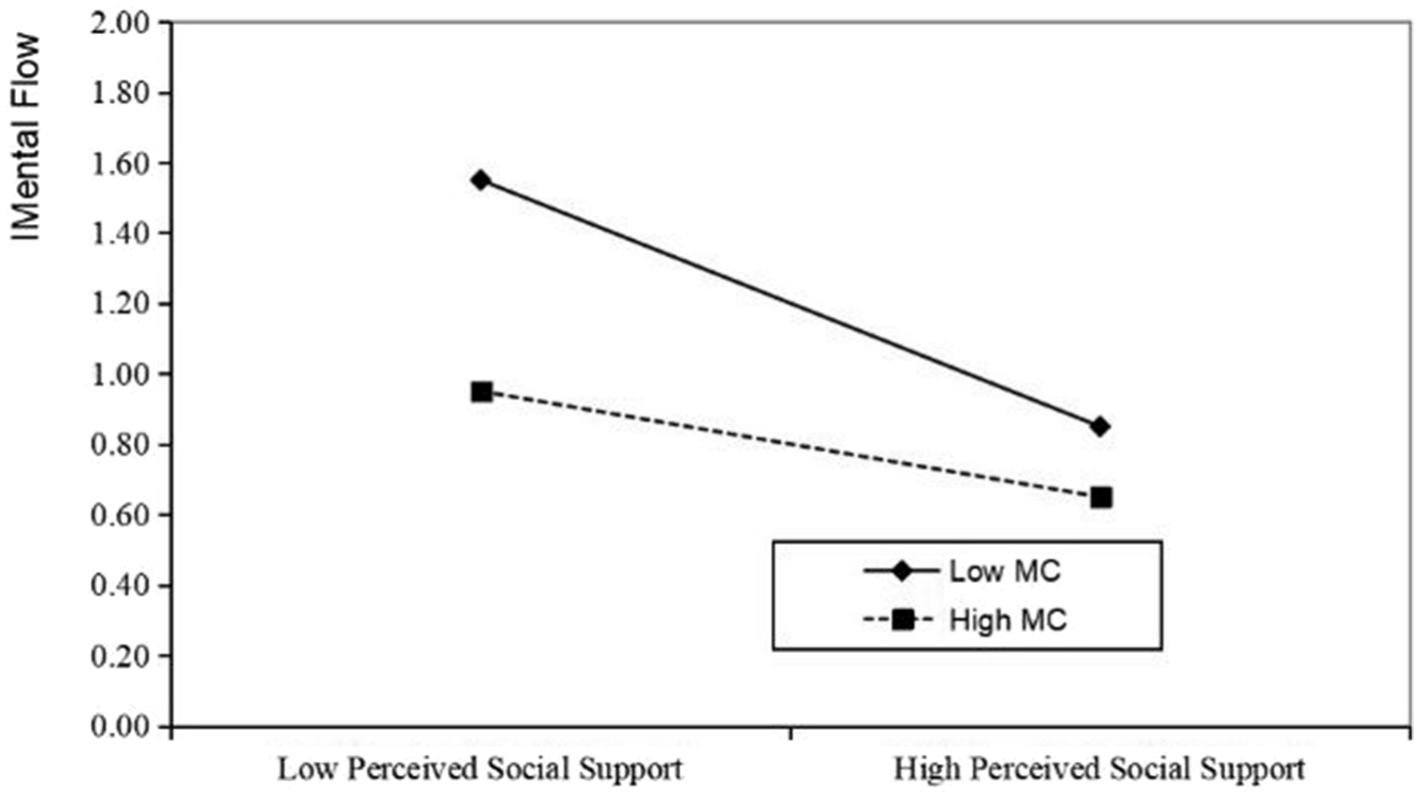
Chained-path moderating effect.

**Figure 5:**
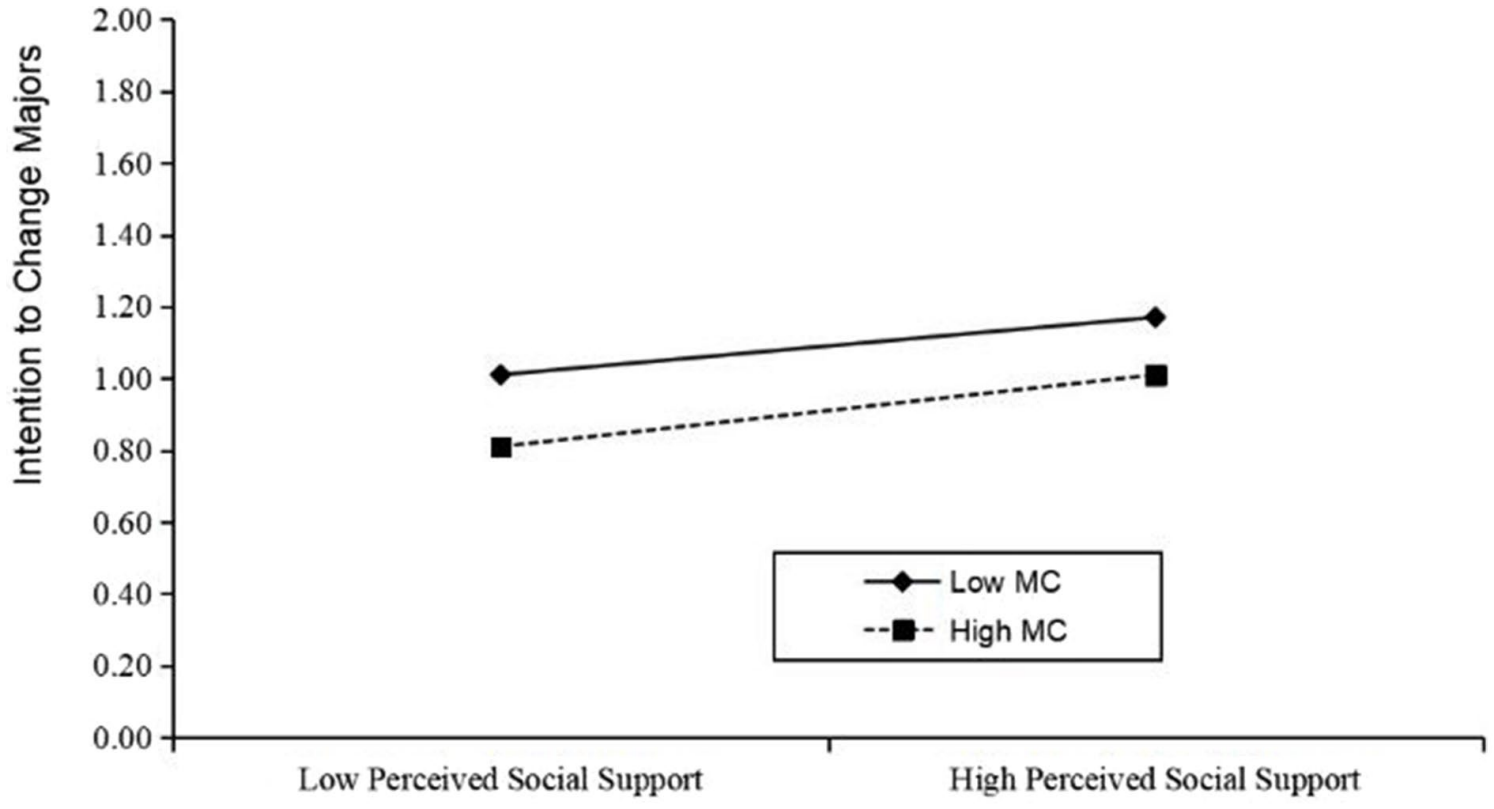
Direct path moderation effect.

In order to more intuitively demonstrate the moderating effect of students’ major cognition on the chain-mediated path of perceived social support and intention to change majors, the variable of students’ major cognition was divided into high and low levels. As can be seen in Figure 4, the addition of major cognition as a moderating variable makes the relationship between perceived social support and students’ mental flow become negatively correlated, and as the level of major cognition increases, it significantly weakens the impact of perceived social support on students’ mental flow. Figure 5 shows that major cognition has no significant moderating effect on the direct relationship between social support and intention to change majors.

## 6 Discussion

The quality of talent development is particularly important for economic and social development. In order to better stimulate students’ enthusiasm for learning and promote the positive growth of high-quality talent, this paper discusses how external environmental factors affect students’ intention to change majors, based on the current interest-oriented major change policy in universities.

First, in analysing the combined effect of students’ perceived social support and their intention to change majors, students’ perceived social support was negatively correlated with their intention to change majors. In the empirical analysis of the data, the results showed that the higher the students’ perceived social support, the lower their intention to change majors. This is consistent with the views of some previous studies (Permatasari et al., 2021; Xin, 2022). The chain mediation effect in the results explains the internal mechanism of the negative correlation between the two. When students can internalise perceived social support, use external resources to achieve the mental flow experience in major learning, experience the fun in major learning, thus stimulating students’ learning self-efficacy, and ultimately reduce the intention to change majors, which achieves the internalisation of external motivation. This is demonstrated by the study by Rumann and Hamrick (2010). The SCCT also points out that individuals are usually in social networks, and the influence of the social environment on individuals plays an important role in individual decision-making (Nguyen et al., 2025).

In addition, the mediating path of self-efficacy as a single mediating variable also proved that if students can translate perceived social support into self-efficacy for major learning, their intention to change majors will also decrease. This is evidenced by the research of Suryaratri et al. (2022), which shows that students are more dependent on the social environment and have stronger emotional needs. Since the total indirect effect is significant, other unmeasured variables have a relatively small impact on the relationship between perceived social support and intention to change majors. From the perspective of individual student attributes, when students are unable to obtain sufficient support or encouragement from the outside world, it can lead to negative emotions such as self-doubt (Guyotte et al., 2019). Students with high self-efficacy are more likely to choose and pursue majors that interest them because they believe they can succeed. This is consistent with the original intention of implementing the major change policy (Wang et al., 2014).

Second, the view that perceived social support increases students’ intention to change majors can also be explained in previous studies. In the results of the study, the direct effect of perceived social support and the intention to change majors showed a significant positive correlation. The higher the level of perceived social support among students, the higher their intention to change majors. The mediating path of the experience of mental flow as a single mediating variable also showed a significant positive correlation. This shows that when students are unable to directly or indirectly translate perceived social support into self-efficacy for major learning, their intention to change majors will increase. This has been defined in previous studies as a lack of independent thinking and perception due to excessive or negative social support, which leads students to passively increase their intention to change majors (Roksa and Kinsley, 2019). For example, students may be forced by their families or given excessive learning pressure by teachers, which leads to passive major changes (Rayle and Chung, 2007). Since there is a lack of intrinsic motivation for students to change majors, this runs counter to the original intention of implementing major change policies. Although a positive correlation between perceived social support and intention to change majors was confirmed, its contribution to total utility was very low. This is because the nature of higher education cultivates students to make independent decisions (Chen et al., 2023), so the positive correlation between the two cannot be taken as the main issue at present. In summary, under different conditions, students’ perceived social support and their intention to change majors are different (Mtshweni, 2024), which supplements and explains the previous research of related scholars.

Finally, students’ major cognition regulates students’ perceived social support and mental flow. Specifically, when students’ major cognition is low, students’ perceived social support has a greater impact on their mental flow of major learning. But when students’ major cognition increases, the effect of social support on students’ mental flow of major learning will weaken or even become negative. The results are consistent with the research findings of Endalcachew et al. (2023), which show that when students have a high level of major cognition, their emotional experience and resilience in the major learning process will increase even when they encounter environmental obstacles. Major cognition, as an important distinguishing point between students’ second and first major choices, is significantly correlated with the level of perceived social support (Yu and Zhang, 2021). Students lack the process of major learning when they first choose a major, so they refer to family, school propaganda, and peer advice and information. However, the intention to change majors generally appears after students have some learning experience. When students have a comprehensive understanding of their major, including information such as the major’s training program and employment status, their goals in the major learning process will become clearer, which will help students improve their mental flow in major learning (Forsblom et al., 2022; Jia and Cheng, 2024). However, it should be noted that the moderating effect of high levels of major cognitive disappears. This is because when major cognitive reaches a high level, an individual’s sense of control over the task, skill match, and focus may already be high enough, and the experience of mental flow is mainly driven by internal factors rather than social support (Bech et al., 2003). Therefore, the role of social support may tend to weaken at high levels of major cognitive. The SCCT mentions that situational variables influence individuals’ career interests and choices by shaping the learning experiences in the SCCT (Lent et al., 1994), which links perceived social support, major cognition, and mental flow. This shows that professional knowledge is an important variable that reflects the influence of students’ self-determination. Therefore, the lack of a significant effect of the major cognitive regulation effect in the direct path also confirms that when students directly adjust their Intention to change majors based on Perceived social support, intrinsic motivation has little effect. In summary, students’ major cognition plays an important role in mediating students’ perceived social support and mental flow in major learning, but the moderating effect is not reflected in the direct effect.

## 7 Implications

The results of this study are of great theoretical and practical significance for increasing students’ enthusiasm for major learning and reducing the risk of major changes. Therefore, based on the research results, this study puts forward the following suggestions.

First, the school should provide students with a good atmosphere for major learning, appropriately get closer to students, and try to avoid excessive restrictive institutional constraints as much as possible on the basis of management principles, so as to create a good environment for major learning, thereby increasing students’ enthusiasm for major learning and reducing their intention to change majors. The sense of pleasure and achievement that students feel during their professional studies is an area that schools should focus on. When students enrol, schools should conduct research on their level of interest in the professional courses and focus on teaching and explaining the courses that are of greater interest.

Second, families and teachers provide positive support and guidance for students. The emotional support, value guidance and behavioural norms provided by families have a profound impact on students’ ability to think for themselves. Teachers, through the imparting of professional knowledge and guidance on learning methods, help students develop an independent way of thinking. Therefore, families and teachers should observe the motivation of students in making a major change. They should support and guide students in making choices based on internal motivation (e.g., major interest), and identify the starting points of external motivation (e.g., conformity, avoidance) and communicate with students to help them transform external motivation into internal motivation. Teachers and families should also observe and guide peer support appropriately to ensure its positivity.

Finally, improve students’ major cognition. Schools should give students a clear understanding of the training programme and employment prospects of their major, and provide students with a clear career development plan, so that students can make a second major choice with a comprehensive understanding of the major. Schools can offer corresponding major cognition and career planning courses to help students improve their major cognition, so that they can better judge their personal professional competence and make decisions about major changes.

The above policy recommendations can minimise the tendency for students to change their major passively other than in an interest-oriented manner, thereby reducing the risk of a weak foundation in major-related learning and errors in choosing a second major brought about by major changes, and ensuring the quality of high-quality talent training.

## 8 Limitations and future research

Although the current study provides innovative findings, several limitations need to be addressed in future studies to enhance the robustness and generalizability of the findings.

First, the geographical limitations of the study may affect the generalizability of the results. This study surveyed five majors in four universities in China, but there are still special factors, such as the degree of leniency of the major transfer policy, cultural differences, and the ease of transfer between majors, that will affect students’ attitudes and behaviours towards major transfer. Future studies should include a wider range of higher education institutions in different regions of various countries, to more thoroughly understand the impact of these regional differences on the research variables.

Second, the cross-sectional design limits the ability to establish a causal relationship between variables. This study examined students’ intention to change majors at a single point in time, and therefore there is a certain degree of error. To address this limitation, future research should use a longitudinal design to track changes over time. The study will track students’ intention to change majors from the time of initial enrolment to the end of the semester to explore changes in students’ intention to change majors as their perception of the major changes. This will more accurately examine the moderation effect and time dynamics of major cognition. In addition, future studies should consider introducing grades as a behavioural variable to investigate the impact of changes in major performance on actual major change behaviour, in order to more accurately reflect students’ major change issues.

Finally, the complete impact mechanism of students’ intention to change majors still needs to be further explored. Previous studies have identified a number of factors that affect the intention to change majors, such as major satisfaction, major commitment and psychological contract. Future research should introduce qualitative interviews and use grounded theory to explore in depth more internal and external factors that affect students’ intention to change majors. At the same time, it is necessary to continue to systematically sort out the mediating variables of perceived social support and intention to change majors, introduce academic performance as a behavioural variable, and study their interrelationships. This approach will clarify the path of association between perceived social support and the intention to change majors, thereby gaining a more comprehensive and detailed understanding of its underlying mechanisms.

## 9 Conclusion

This paper adopts the SCCT framework to study the relationship between students’ perceived social support, mental flow experience, self-efficacy, major cognition and major mobility from the perspective of the mutual transformation between open and closed environments. Specifically, this paper aims to explore how perceived social support affects students’ intention to change majors through the mediating effects of mental flow experience and self-efficacy, as well as the moderating effect of major cognition. The paper finds that students’ mental flow experience and self-efficacy have a chain mediation effect on the relationship between perceived social support and intention to change majors. In addition, students’ major cognition significantly moderated the chain mediation path between perceived social support and intention to change majors. The SCCT emphasises that individuals usually convert external factors into internal motivation to influence behavioural intentions. Perceived social support, as a complex external environmental factor, can only reduce students’ intention to change majors when it enhances their self-efficacy in their chosen major. Otherwise, students are more likely to give up their current major. In addition, attention should also be paid to students’ major cognition, because a high level of major cognition can enhance students’ ability to make independent decisions, thereby reducing the risk of major changes. The paper used a chain mediation effect analysis to analyse the impact mechanism in more detail, which is more convincing at the empirical level than a simple mediation effect analysis and linear regression, and provides a more favourable reference basis for policy formulation. The main findings of this paper provide new insights into college students’ major selection and offer targeted policy recommendations for reducing the risk of changing majors, enhancing students’ learning enthusiasm, and strengthening the cultivation of high-quality talent.

## Data Availability

The raw data supporting the conclusions of this article will be made available by the authors, without undue reservation.
